# Isotretinoin and serum thyroid parameters: systematic review and meta-analysis^؆^

**DOI:** 10.1016/j.abd.2026.501380

**Published:** 2026-06-03

**Authors:** Niyaz Mostafa, Esther Hong, Thomas Stewart

**Affiliations:** a St George Dermatology and Skin Cancer Centre, Sydney, NSW, Australia; b Department of Dermatology, Royal North Shore Hospital, Sydney, NSW, Australia; c Faculty of Medicine, The University of Sydney, Sydney, NSW, Australia

**Keywords:** Acne vulgaris, Autoantibodies, Hypothyroidism, Isotretinoin, Systematic review, Thyroid function tests, Thyroid hormones

## Abstract

**Background:**

Isotretinoin is an effective therapy for acne vulgaris, but its impact on thyroid function remains uncertain, with existing studies reporting inconsistent biochemical changes.

**Objective:**

To assess the effects of oral isotretinoin on serum thyroid function parameters and thyroid antibody levels in patients with acne vulgaris.

**Methods:**

A systematic review and meta-analysis of observational studies was conducted according to PRISMA guidelines and prospectively registered (INPLASY202560049). PubMed, Scopus, Embase, and Web of Science were searched from January 1980 to September 2025. Studies reporting serum thyroid parameters before and during isotretinoin therapy were included. Random-effects meta-analyses were performed for parameters reported in more than three studies. Heterogeneity was assessed using the I^2^ statistic, and risk of bias using NIH tools.

**Results:**

Fifteen studies were included. Meta-analysis showed a small statistically significant increase in thyroid-stimulating hormone after isotretinoin treatment (MD = 0.03, 95% CI 0.02‒0.05, p < 0.001), with significant decreases in thyroxine (MD = -0.19, 95% CI −0.24 to −0.15, p < 0.001) and triiodothyronine (MD = -0.62, 95% CI-0.76 ‒ -0.48, p < 0.001). Heterogeneity was substantial for thyroid-stimulating hormone (I^2^ = 85%) and triiodothyronine (I^2^ = 86%), and moderate for thyroxine (I^2^ = 60%). Evidence regarding thyroid antibodies was limited and inconsistent.

**Study limitations:**

Included studies were observational, predominantly single-centre, and heterogeneous in dosing, duration, and laboratory assessments.

**Conclusions:**

Isotretinoin is associated with statistically significant alterations in serum thyroid function parameters, though the clinical significance of these changes remains uncertain.

## Introduction

Isotretinoin has been used in the treatment of acne vulgaris and other follicular occlusion disorders since the 1980s and remains an effective therapy for severe or refractory acne. Acne vulgaris is one of the most common dermatological conditions worldwide, with an estimated global prevalence of 9.4%. Acne is more common in adolescents; however also affects a substantial proportion of adults.^1^ The condition can lead to significant psychosocial morbidity and permanent scarring, highlighting the importance of effective treatment strategies.^2^ Isotretinoin’s mechanism of action remains incompletely understood but is thought to be mediated through effects on sebum production, hyperkeratinization, inflammation, and Cutibacterium acnes.^3^ It has been postulated that one of the ways in which isotretinoin may help to treat acne is by suppressing pituitary hormone levels.^4^, ^5^

Despite its efficacy, isotretinoin therapy is associated with a wide range of adverse effects that require clinical and biochemical monitoring. Several systematic reviews and meta-analyses have evaluated the safety profile of isotretinoin and consistently report laboratory alterations involving lipid metabolism, liver function tests, and hematological parameters. For example, systematic reviews have shown that isotretinoin therapy may lead to increases in serum triglycerides, cholesterol, and low-density lipoprotein levels, with variable effects on liver enzymes and other laboratory markers.^6^, ^7^

In addition to metabolic effects, isotretinoin has also been implicated in endocrine changes. Retinoids interact with nuclear receptors that belong to the steroid-thyroid hormone receptor superfamily, suggesting that isotretinoin may influence hormonal signalling pathways, including those involving the hypothalamic-pituitary-thyroid axis. Some observational studies have reported alterations in thyroid function tests during isotretinoin therapy, including increases in Thyroid-Stimulating Hormone (TSH) and reductions in circulating Triiodothyronine (T3) and Thyroxine (T4).^5^ Additionally, acne itself has been associated with endocrine abnormalities, including hypothyroidism and autoimmune thyroiditis, further complicating the interpretation of these biochemical findings.^8^

Although individual studies have explored the relationship between isotretinoin and thyroid function, the data remain limited and conflicting. Furthermore, while previous systematic reviews investigating the use of isotretinoin in acne have examined its other systemic adverse effects, to date, there has been limited prior synthesis of the available evidence specifically addressing thyroid function during isotretinoin therapy.^6^, ^7^

Given the widespread use of isotretinoin and the potential systemic implications of thyroid dysfunction, clarification of this relationship is clinically important. A comprehensive synthesis of existing evidence may help determine whether changes in thyroid parameters represent a consistent pharmacological effect of isotretinoin or reflect heterogeneity between individual studies.

Therefore, the aim of this systematic review and meta-analysis is to collate and describe observational studies of serum thyroid function and antibody testing in patients taking oral isotretinoin and to evaluate the effects of oral isotretinoin therapy on serum thyroid function parameters and thyroid antibody levels in patients undergoing treatment for acne vulgaris.

## Methods

This systematic review was conducted using PRISMA (Preferred Reporting Items for Systematic reviews and Meta-Analyses) guidelines and prospectively registered with INPLASY (International Platform of Registered Systematic Review and Meta-analysis Protocols) with registration number INPLASY202560049.

The review question was formulated using the Population-Exposure-Outcome (PEO) framework. The population comprised individuals of any age or sex receiving oral isotretinoin therapy, primarily for acne vulgaris. The exposure was treatment with systemic isotretinoin. The outcomes of interest were changes in serum thyroid function parameters, including Thyroid-Stimulating Hormone (TSH), Thyroxine (T4), Triiodothyronine (T3), and thyroid antibody levels.

Eligible study designs included observational studies reporting thyroid function measurements before and during isotretinoin therapy. These included cohort studies, case-control studies, observational studies, and case series. Studies conducted in human participants and reporting at least one thyroid parameter were considered for inclusion.

### Data sources

The information sources for this review encompassed PubMed (January 1, 1980 ‒ September 5, 2025), Scopus (January 1, 1980 ‒ September 5, 2025), Embase (January 1, 1980 ‒ September 5, 2025), and Web of Science (January 1, 1980 ‒ September 5, 2025). The full search strategy for PubMed, including Boolean operators and filters applied, is presented in Table 1.

**Table 1:** Search strategy.

**Resources:**
1) Pubmed (January 1, 1980 – September 5, 2025)
2) Scopus (January 1, 1980 – September 5, 2025)
3) Europe PMC (January 1, 1980 – September 5, 2025)
4) Embase (January 1, 1980 – September 5, 2025)
**Full Search Strategy (PubMed)**
Isotretinoin OR Roaccutane OR Accutane OR 13-cis-retinoic acid AND thyroid hormone OR thyroid-stimulating hormone OR thyroxine OR triiodothyronine OR thyroid antibodies OR thyroglobulin antibodies OR thyroid peroxidase antibodies OR thyroid-stimulating hormone receptor antibodies OR thyrotropin receptor antibodies
**Filters applied:**
Humans; English language; publication date from 1 January 1980 to 5 September 2025.

### Study eligibility criteria

The eligibility criteria for this review included observational studies, such as cohort studies, case-control studies, and case series. There are no restrictions placed on age, sex or ethnicity.

Eligible studies were those reporting at least one thyroid parameter (thyroid-stimulating hormone, thyroxine, triiodothyronine, thyroid peroxide antibodies, thyroglobulin antibodies, thyroid-stimulating hormone receptor antibodies, thyrotropin receptor antibodies) tested in the serum of subjects taking oral isotretinoin.

Articles deemed not eligible were those which: Reported results from animal (non-human) subjects; Were written in a language other than English; Were reports of single cases.

### Study selection

**Fig. 1:**
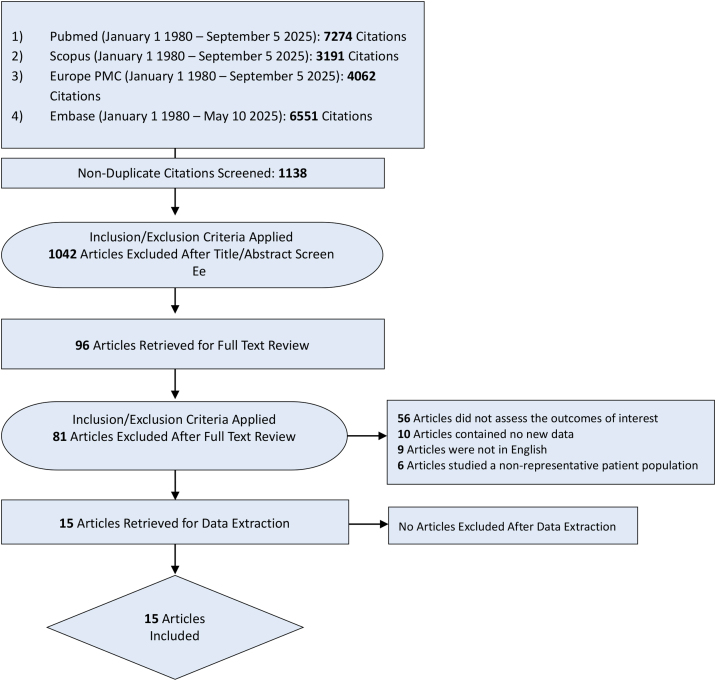
PRISMA flowchart of search results.

All records identified through the database searches were imported into a reference management software, and duplicates were removed. Two reviewers independently screened titles and abstracts for relevance. Articles that did not meet the inclusion criteria were excluded at this stage. The full texts of potentially eligible studies were then assessed independently by two reviewers. Any disagreements regarding study inclusion were resolved through discussion with a third reviewer. The study selection process is summarized in the PRISMA flow diagram (Fig. 1).

### Appraisal and synthesis methods

Data collection was performed independently by two authors, with any disagreements regarding data being referred to a third author for mediation. Information was collected using a standardised data collection form, with the principal outcomes of interest being serum thyroid parameters. If data from individual patients was not available, then the aggregate data were collected. Potential sources of bias in the identified studies are acknowledged, including the small size of patient cohorts and the variability in the methods and scope of testing. Therefore, these variables were collated to assess the heterogeneity of studies. Bias was assessed using the NIH quality assessment tools (Supplementary Table S1‒S2).

### Meta-analysis

Following full-text screening, all fifteen studies were deemed eligible for data extraction. The sample size for cases reporting serum thyroid parameter/s was pooled and parameters reported in more than three studies were subjected to quantitative analysis. Statistical analyses were performed with RStudio 4.3.1 (RStudio: Integrated Development for R. RStudio, PBC, Boston, MA URL http://www.rstudio.com/) using packages *meta 6.5-0* and *dmetar.* Meta-analysis for mean differences was performed with the *metaprop* function and presented as a Forest plot. A Funnel plot was constructed to make a visual assessment of whether small-study effects were present and used to assess publication bias.

## Results

Fifteen articles were eligible for inclusion (Table 2).^4^, ^5^, ^8^, ^9^, ^10^, ^11^, ^12^, ^13^, ^14^, ^15^, ^16^, ^17^, ^18^, ^19^, ^20^ Twelve studies were cohort designs and three were case-control designs. Six studies included patients under the age of eighteen years, with the youngest aged fourteen years. Two studies included females only, and a single study included males only. All studies, except one, were conducted on individuals with acne vulgaris. All studies except one used weight-based dosing, with the lowest and highest being 0.2 mg/kg/day and 0.8 mg/kg twice daily, respectively. Six studies employed a dosing range. The most common treatment duration was three months, with a maximum of eight months. All fifteen articles reported on Thyroid-Stimulating Hormone (TSH) levels, thirteen measured Thyroxine (T4), and twelve measured Triiodothyronine (T3). Three articles reported thyroid antibodies. Serum thyroid testing was done prior to initiation of isotretinoin and on at least one other occasion in all studies, with an upper limit of eight months.

**Table 2:** Study demographics.^4^, ^5^, ^8^, ^9^, ^10^, ^11^, ^12^, ^13^, ^14^, ^15^, ^16^, ^17^, ^18^, ^19^, ^20^

First author/Year	Country	Study design	Population	N =	Thyroid parameters tested
Ahmed/2021^5^	Egypt	Cohort	Acne vulgaris	50 (16‒61 years)	TSH, T4, T3
Aktar/2020^8^	Turkey	Case control	Acne vulgaris	60 (18‒40 years) (female only)	TSH
AlSaif/2020^9^	Saudi Arabia	Prospective cohort	Acne vulgaris	51 (18‒25 years)	TSH, T4, TrAbs
Chandrakar/2022^10^	India	Cohort	Acne vulgaris	100 (18‒40 years)	TSH
Hareedy/2021^11^	Egypt	Cohort	Acne vulgaris	47 (16‒29 years)	TSH, T4, T3
Karadag/2011^4^	Turkey	Cohort	Acne vulgaris	47 (17‒34 years)	TSH, T4, T3, TrAbs
Karadag/2015^12^	Turkey	Cohort	Acne vulgaris	105 (14‒42 years)	TSH, T4, T3
Kocyigit/2020^13^	Turkey	Case control	Acne vulgaris	30 (18‒45 years) (female only)	TSH, T4, T3
Kotb/2025^14^	Saudi Arabia	Prospective cohort	Acne Vulgaris	50 (18‒35 years)	TSH, T4, T3
Lyons/1982^15^	UK	Cohort	Acne vulgaris	18 (17‒42 years)	TSH, T4, T3
Marsden/1984^16^	UK	Cohort	Rosacea	7 (29‒60 years)	TSH, T4, T3
Morey/2020^17^	India	Cohort	Acne vulgaris	30 (15‒30 years)	TSH, T4, T3
O’Leary/1986^18^	Canada	Cohort	Acne vulgaris	24 (male only)	TSH, T4, T3
Uyar/2016^19^	Turkey	Case control	Acne vulgaris	66 (18‒39 years)	TSH, T4, T3, TrAbs
Yildirim/2017^20^	Turkey	Cohort	Acne vulgaris	51 (18‒32 years)	TSH, T4, T3

Notes: TSH, Thyroid-Stimulating Hormone, T3, Triiodothyronine, T4, Thyroxine, TrAbs, Thyroid Antibodies.

T4 and T3 levels were shown to have decreased after isotretinoin treatment in nine and eight studies, respectively. TSH levels were found to have increased and remained unchanged in seven and six studies, respectively. Thyroid antibodies were found in two studies to have decreased, and remained unchanged in one. Isotretinoin and serum thyroid parameters are summarized in Table 3.

**Table 3:** Isotretinoin and serum thyroid parameters.^4^, ^5^, ^8^, ^9^, ^10^, ^11^, ^12^, ^13^, ^14^, ^15^, ^16^, ^17^, ^18^, ^19^, ^20^

First author/Year	Study population	Dose of isotretinoin	Duration of isotretinoin	Testing intervals	Thyroid parameter results
Ahmed/2021 ^5^	Children and adults with acne vulgaris	0.5‒0.8 mg/kg/day	At least 3-months	Pre-treatment and 3 months	TSH increased after treatment
					T3 decreased after treatment
					T4 decreased after treatment
Aktar/2020 ^8^	Adult females with acne vulgaris	0.5 mg/kg/day	3-months	Pre-treatment and 3 months	TSH showed no difference after treatment
AlSaif/2020 ^9^	Adults with acne vulgaris	0.5 mg/kg/day	8-months	Pre-treatment, months 2, 4, 6 and 8	TSH showed no difference after treatment
					T4 showed no difference after treatment
					TrABs showed no difference after treatment
Chandrakar/2022 ^10^	Adults with acne vulgaris	20 mg/day	4-months	Pre-treatment and 4 months	TSH increased after treatment
Hareedy/2021 ^11^	Adults with acne vulgaris	0.5 mg/kg/day	6-months	Pre-treatment, 3 months and 6 months	TSH increased after treatment
					T3 decreased after treatment
					T4 decreased after treatment
Karadag/2011 ^4^	Young adults with acne vulgaris	0.5‒0.75 mg/kg twice daily	At least 5-months	Pre-treatment and 3 months	TSH decreased after treatment
					T3 decreased after treatment
					T4 showed no difference after treatment
					TrABs decreased after treatment
Karadag/2015^12^	Children and adults with acne vulgaris	0.5‒1 mg/kg/day	3 months	Pre-treatment and 3 months	TSH showed no difference after treatment
		0.2‒0.5 mg/kg/day			T3 decreased after treatment
		0.5‒1 mg/kg/day 1-week/month			T4 (high dose) decreased after treatment
Kocyigit/2020^13^	Adult women with acne vulgaris	120‒150 mg/kg (total dose)	3-months	Pre-treatment and 3 months	TSH increased after treatment
					T3 showed no difference after treatment
					T4 decreased after treatment
Kotb/2025^14^	Adults with acne vulgaris	0.5‒1 mg/kg twice daily	At least 4-months	Pre-treatment and 4 months	TSH increased after treatment
					T3 decreased after treatment
					T4 decreased after treatment
Lyons/1982^15^	Children and adults with acne vulgaris	0.8 mg/kg/day	3-months	Pre-treatment, 3 months, 4 months	TSH showed no difference after treatment
					T3 showed no difference after treatment
					T4 decreased after treatment
Marsden/1984^16^	Adults with rosacea	1 mg/kg/day	3-months	Pre-treatment, 6-weeks, 3-months and 4-months	TSH showed no difference after treatment
					T3 decreased after treatment
					T4 decreased after treatment
Morey/2020^17^	Children and adults with acne vulgaris	0.5 mg/kg/day	3-months	Pre-treatment and 3-months	TSH decreased after treatment
					T3 increased after treatment
					T4 increased after treatment
O’Leary/1986^18^	Adult males with acne vulgaris	1 mg/kg/day	4-months	Pre-treatment, weeks 4, 8, 12 and 16	TSH showed no difference after treatment
					T3 showed no difference after treatment
					T4 showed no difference after treatment
Uyar/2016^19^	Adults with acne vulgaris	0.5‒0.8 mg/kg/day twice daily	At least 4-months	Pre-treatment and 4-months	TSH increased after treatment
					T3 decreased after treatment
					T4 decreased after treatment
					TrABs decreased after treatment
Yildirim/2017^20^	Young adults with acne vulgaris	0.5‒1 mg.kg/day	Up to 6-months	Pre-treatment, 3-months and 6-months	TSH increased after treatment
					T3 decreased after treatment
					T4 decreased after treatment

Notes: TSH, Thyroid-Stimulating Hormone; T3, Triiodothyronine; T4, Thyroxine; TrAbs, Thyroid Antibodies; NR, Not Reported.

### Meta-analysis

**Fig. 2:**
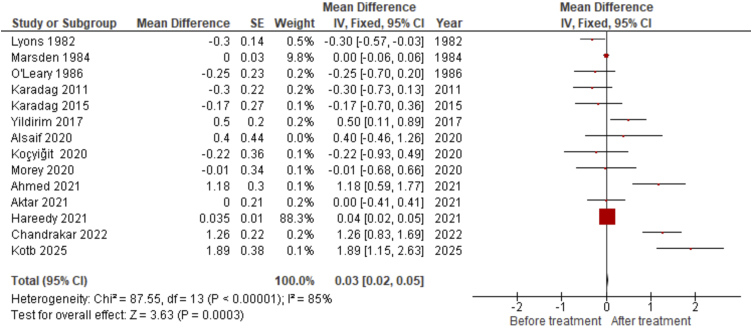
Forest plot of studies reporting TSH levels.^4^, ^5^, ^8^, ^9^, ^10^, ^11^, ^12^, ^13^, ^14^, ^15^, ^16^, ^17^, ^18^, ^20^

**Fig. 3:**
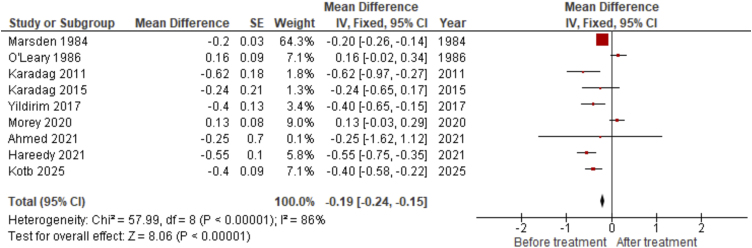
Forest plot of studies reporting Triiodothyronine (T3) levels.^4^, ^5^, ^11^, ^12^^,^^14^, ^16^, ^17^, ^18^, ^20^

**Fig. 4:**
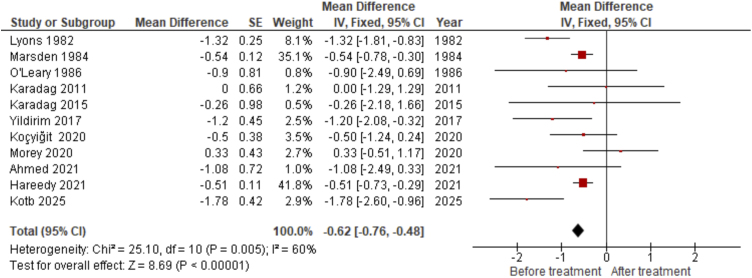
Forest Plot of studies reporting Thyroxine (T4) levels.^4^, ^5^, ^11^, ^12^, ^13^, ^14^, ^15^, ^16^, ^17^, ^18^, ^20^

Pooled results showed a small, but statistically significant increase in the levels of thyroid-stimulating hormone (Mean Difference [MD = 0.03], 95% CI 0.02‒0.05, p < 0.001) following the administration of isotretinoin (Fig. 2). Conversely, there was a statistically significant decrease in the levels of both thyroxine (MD = −0.19, 95% CI −0.24 to −0.15, p < 0.001) and triiodothyronine (MD = −0.62, 95% CI −0.76 to −0.48, p < 0.001) (Fig. 3, Fig. 4).

### Assessment of study heterogeneity

The heterogeneity between the studies reporting TSH and T3 levels was substantial (I^2^ = 85% and 86%, respectively). There was moderate heterogeneity between studies reporting thyroxine (I^2^ = 60%). There were 12 cohort studies and three case-control studies and all 15 were sampled from a single centre. The sample size ranged widely (7 to 100) and three studies sampled a single sex only. Four and seven different thyroid panels and testing intervals were reported, respectively. The dose of isotretinoin varied between the lowest and highest by 1.4 mg/kg/day, and duration by months.

### Assessment of bias

The results of bias assessments using NIH criteria are presented in Supplementary Tables S1 and S2. All 15 studies clearly stated the research question and had well-defined study populations. The subjects (and controls) in all 15 studies were selected from the same/similar populations; however, only nine cohort studies documented inclusion and/or exclusion criteria. Karadag et al. was the only study to consider different levels of exposure.^13^ None of the case control studies documented having blinded their assessors to the case or control status of the participants. Kocyigit et al. was the only study to account for possible confounding variables.^14^ Funnel plots constructed using studies reporting thyroid-stimulating hormone, thyroxine and triiodothyronine all showed low risk of small study effect and publication bias (Supplementary Figs. S1‒S3).

## Discussion

This systematic review and meta-analysis synthesizes the available evidence regarding the effects of oral isotretinoin on thyroid function and autoimmunity in the acne vulgaris population. Across fifteen studies, isotretinoin therapy was associated with a statistically significant increase in thyroid-stimulating hormone and reductions in both Thyroxine (T4) and Triiodothyronine (T3).^4^, ^5^, ^8^, ^9^, ^10^, ^11^, ^12^, ^13^, ^14^, ^15^, ^16^, ^17^, ^18^, ^19^, ^20^ These findings suggest that isotretinoin may exert measurable effects on the hypothalamic-pituitary-thyroid axis, although the clinical significance of these changes requires cautious interpretation.

From a clinical perspective, the magnitude of thyroid hormone changes reported in most studies appears modest and frequently remains within reference ranges.^9^, ^16^, ^17^, ^19^ This suggests that, for the majority of patients, isotretinoin is unlikely to exert clinically-apparent thyroid dysfunction. Nevertheless, the consistent direction of change across multiple studies, characterized by increased TSH and reduced T3 and T4, raises the possibility of a mild, subclinical alteration in thyroid homeostasis on treatment. Similar biochemical alterations have been described in previous studies evaluating thyroid function during isotretinoin therapy.^5^, ^11^, ^20^

Several biological mechanisms may explain these findings. Retinoids act through nuclear retinoic acid receptors and Retinoid X Receptors (RXRs), which belong to the steroid-thyroid hormone receptor superfamily.^21^ Importantly, RXRs can form heterodimers with thyroid hormone receptors and regulate transcription of thyroid hormone-responsive genes, providing a mechanistic explanation for the interaction between retinoid signaling and thyroid hormone pathways.^22^ Through these shared nuclear receptor pathways, isotretinoin or its metabolites may influence thyroid hormone synthesis, metabolism, and/or pituitary regulation of TSH secretion.

Although these biochemical changes appear largely subclinical, the findings may be most relevant to selected populations. Individuals with pre-existing thyroid disease or borderline thyroid function may be more susceptible to hormonal perturbations during isotretinoin treatment. Retinoids have also been implicated in immune modulation and endocrine signaling, suggesting a potential role in modifying thyroid autoimmunity in susceptible individuals, although evidence remains limited.^23^, ^24^ In clinical practice, this raises the possibility that patients who develop symptoms suggestive of thyroid dysfunction (e.g. fatigue, weight changes, mood disturbance) on isotretinoin treatment may benefit from targeted biochemical evaluation.

Interpretation of these findings must also consider the substantial heterogeneity observed between included studies. Differences in isotretinoin dosing regimens, treatment duration, study populations, and laboratory testing intervals likely contributed to the variability in reported outcomes. Additionally, thyroid antibody testing was performed in only a small subset of studies, limiting conclusions regarding the potential effects of isotretinoin on thyroid autoimmunity.

Overall, this review suggests that isotretinoin therapy is associated with consistent but generally mild alterations in thyroid parameters that are unlikely to be clinically significant in most patients. However, the findings highlight a potentially under-recognized endocrine effect of isotretinoin and suggest that targeted thyroid monitoring may be appropriate in selected individuals. Larger prospective studies with standardized thyroid testing protocols and longer follow-up will be useful to clarify whether these biochemical changes translate into clinically significant thyroid dysfunction.

## Limitations

This review had several limitations that must be considered when interpreting the findings.

All studies were observational in design, with most being small, single-centre cohorts, which possibly introduced selection bias. There was considerable heterogeneity between the studies, reflected in dosing regimens, treatment duration, and laboratory testing methods. This compromises the pooling of results and limits the comparability of effect sizes. Only a single study measured different levels of exposure, which can lead to inaccurate categorisation of measured exposures, resulting in an incorrect estimate of the exposure’s effect. Generally, the studies did not adjust for possible confounding factors, with very few reporting actively excluding participants with pre-existing thyroid disease or asking about participants' other regular medications. It is also possible that publication bias played a role in our sample, as we restricted our search to the English language and only three of the included studies reported ‘no effect’ across their measured parameters.

## Conclusions/Recommendations

This systematic review and meta-analysis suggest that isotretinoin use is associated with changes in serum thyroid function parameters, with the overall trend towards increased TSH and decreased triiodothyronine and thyroxine. Future research should include prospective, multicenter studies with rigorous inclusion and exclusion criteria, standardized testing panels (including thyroid antibodies), multivariate analyses, and long-term follow-up to better elucidate the clinical significance and durability of these isotretinoin-induced thyroid alterations.

## Financial support

None declared.

## Authors' contributions

Niyaz Mostafa: The study concept and design; data collection, or analysis and interpretation of data; statistical analysis; writing of the manuscript or critical review of important intellectual content; data collection, analysis, and interpretation; effective participation in the research guidance; intellectual participation in the propaedeutic and/or therapeutic conduct of the studied cases; critical review of the literature; final approval of the final version of the manuscript.

Esther Hong: The study concept and design; data collection, or analysis and interpretation of data; statistical analysis; writing of the manuscript or critical review of important intellectual content; data collection, analysis, and interpretation; effective participation in the research guidance; intellectual participation in the propaedeutic and/or therapeutic conduct of the studied cases; critical review of the literature; final approval of the final version of the manuscript.

Thomas Stewart: The study concept and design; data collection, or analysis and interpretation of data; statistical analysis; writing of the manuscript or critical review of important intellectual content; data collection, analysis, and interpretation; effective participation in the research guidance; intellectual participation in the propaedeutic and/or therapeutic conduct of the studied cases; critical review of the literature; final approval of the final version of the manuscript.

## ORCID ID

Esther Hong: 0009-0006-0046-2485

Thomas Stewart: 0000-0001-7407-2857

## Research data availability

The entire dataset supporting the results of this study was published in this article.

## Conflicts of interest

None declared.
