# Supplementary material for: Isotretinoin and serum thyroid parameters: systematic review and meta-analysis
Source: An Bras Dermatol. 2026 Jun 3;101(4):501380. doi: 10.1016/j.abd.2026.501380 (PMC13265679; doi:10.1016/j.abd.2026.501380)
Supplement: Supplementary file 1 [file mmc1.docx]

ABD-D-26-00075_Supplementary Material

**Supplementary Table 1** NIH bias assessment of cohort studies.[4-5,9-12,14-18,20]

| **First Author/year** | **Was the research question or objective clearly stated?** | **Was the study population clearly specified and defined?** | **Was participation at least 50%?** | **Were all the subjects selected from the same/ similar population? Were inclusion & exclusion criteria prespecified and applied uniformly?** | **Was a sample size justification, power description, or variance and effect estimates provided?** | **Were the exposures of interest measured prior to the outcome(s) being measured?** | **Was the timeframe sufficient to reasonably expect to see an association between exposure and outcome if it existed?** | **Did the study examine different levels of the exposure as related to the outcome?** | **Were the exposures clearly defined, valid, reliable & implement-ted consistently across all study participants?** | **Was the exposure assessed more than once over time?** | **Were the outcome measures clearly defined, valid, reliable & implement-ted consistently across all participants?** | **Were the outcome assessors blinded to the exposure status of participants?** | **Was loss to follow-up after baseline 20% or less?** | **Were confounding variables measured & adjusted statistically for their impact on the relationship between exposures & outcomes?** |
| --- | --- | --- | --- | --- | --- | --- | --- | --- | --- | --- | --- | --- | --- | --- |
| Ahmed/2021 | Y | Y | NR | Y/Y | N | Y | Y | N | Y | N | Y | N | Y | N |
| AlSaif/2020 | Y | Y | Y | Y/Y | N | Y | Y | N | Y | N | Y | N | Y | N |
| Chandrakar/2022 | Y | Y | Y | Y/Y | N | Y | Y | N | Y | N | Y | N | Y | N |
| Hareedy/2021 | Y | Y | Y | Y/Y | N | Y | Y | N | Y | N | Y | N | Y | N |
| Karadag/2011 | Y | Y | Y | Y/Y | N | Y | Y | N | Y | N | Y | N | Y | N |
| Karadag/2015 | Y | Y | Y | Y/Y | N | Y | Y | Y | Y | N | Y | N | Y | N |
| Kotb/2025 | Y | Y | Y | Y/Y | N | Y | Y | N | Y | N | Y | N | Y | N |
| Lyons/1982 | Y | Y | NR | Y/N | N | Y | Y | N | Y | N | Y | N | Y | N |
| Marsden/1984 | Y | Y | NR | Y/N | N | Y | Y | N | Y | N | Y | N | Y | N |
| Morey/2020 | Y | Y | Y | Y/Y | N | Y | Y | N | Y | N | Y | N | Y | N |
| O‘Leary/1986 | Y | Y | NR | Y/N | N | Y | Y | N | Y | N | Y | N | Y | N |
| Yilidrim/2017 | Y | Y | NR | Y/Y | N | Y | Y | N | Y | N | Y | N | Y | N |

Notes: NR, Not Reported.

**Supplementary Table 2** NIH bias assessment of case control studies.[8,13,19]

| **First author/year** | **Was the research question or objective clearly stated and appropriate?** | **Was the study population clearly specified and defined?** | **Did the authors include a sample size justification?** | **Were controls selected or recruited from the same or similar population that gave rise to the cases?** | **Were the definitions, inclusion and exclusion criteria, algorithms or processes used to select cases and controls valid, reliable & implemented consistently across all study participants?** | **Were the cases clearly defined and differentiated from controls?** | **If less than 100% of eligible cases and/or controls were selected for the study, were the cases and/or controls randomly selected from those eligible?** | **Was there use of concurrent controls?** | **Were the investigators able to confirm that the exposure/risk occurred prior to the development of the condition or event that defined a participant as a case?** | **Were the measures of exposure/risk clearly defined, valid, reliable, and implemented consistently (including time period) across all study participants?** | **Were the assessors of exposure/risk blinded to the case or control status of participants?** | **Were key potential confounding variables measured and adjusted statistically in the analyses? If matching was used, did the investigators account for matching during study analysis?** |
| --- | --- | --- | --- | --- | --- | --- | --- | --- | --- | --- | --- | --- |
| Aktar/2020 | Y | Y | N | Y | Y | Y | NS | Y | Y | Y | N | N |
| Kocyigit/2020 | Y | Y | N | Y | Y | Y | NS | Y | Y | Y | N | Y |
| Uyar/2016 | Y | Y | N | Y | Y | Y | NS | Y | Y | Y | N | N |

NS, Not Specified.

**Supplementary Figure 1** Funnel plot of studies reporting TSH levels.[4-5,8-18,20]


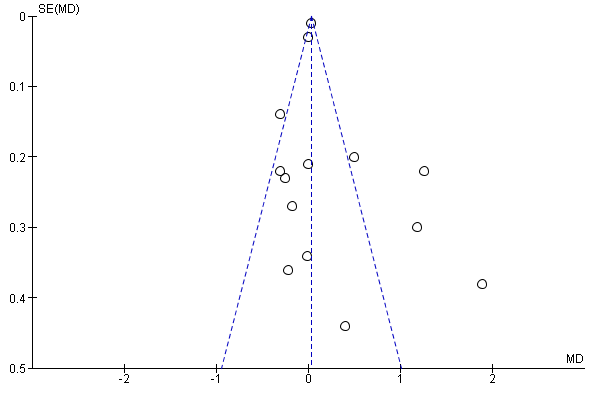


**Supplementary Figure 2** Funnel plot of studies reporting Triiodothyronine (T3) levels.[4-5,11-12,14,16-18,20]


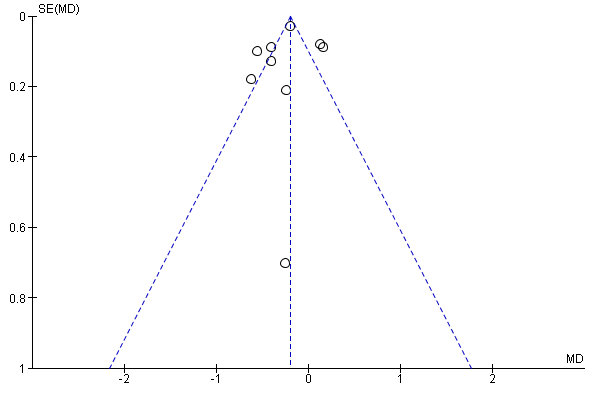


**Supplementary Figure 3** Funnel plot of studies reporting Thyroxine (T4) levels.[4-5, 11-18,20]

**
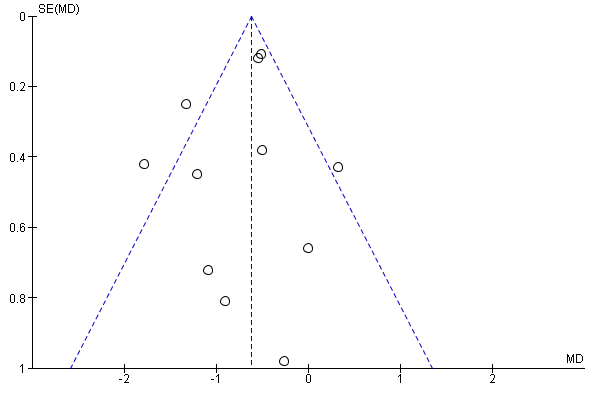
**
